# Everybody's Page

**Published:** 1892-09-17

**Authors:** 


### Everybody's Page.

Anti-vaccinationists should rejoice that they do not live in a country so oppressed by vaccination laws as Italy. In that country at the beginning of the year a new law came into effect which renders not only vaccination, but re-vaccination necessary. Children must be vaccinated before they are six months old, and again at eight years. Those who have not complied with the regulations are excluded from schools, factories, benevolent institutions, &c.

---

A story of interest is told relating to a distinguished German physiologist, the late Professor Bischoff. He was a firm believer in the intellectual inferiority of woman. This he attributed to the fact that the female brain was of lighter weight than the male; the former, he determined, after long research, to average 1,350 grammes, and the latter only 1,250 grammes. Unfortunately for the Professor's reputation, if judged according to his own lights, his brain was weighed after death, and found to be only 1,245 grammes in weight.

---

What is the limit to what modern thought will evolve from the past? Who could have imagined that Luther should ever be accused of being at the root of the German Socialism of to-day! But so we have it from a French pen, printed in the Paris "Revue des Revues." From his writings, we are told, an abundant harvest of Socialistic ideas are to be gathered. The ruin of princes and tyrants is predicted. Kant, Fichte, and Hegel are stated to have finished sowing the seed commenced by Luther.

---

What would our English students say if at our universities the rules respecting athletics at Harvard were in force? There the university authorities have arrived at the conclusion that whilst exercise in moderation not only does not impede, but actually helps brain workers, they do not believe in making athletics the end and object of a young man's career. To prevent this, no student is permitted to participate in inter-collegiate sports, unless he maintains a certain standing in his studies. The junior members of our universities would resent such a restriction as this in the highest degree; especially would it be unpopular at those colleges, known as "good boating" or "football" colleges, and which owe their popularity literally to their athletic reputations.

---

An endeavour is being made to prove a connection between sun spots and insanity. In other words, the suggestion is made that insanity is heliocy rather than lunacy. The opinion first arose from observations of sudden cases of insanity during the sun-spot maximum from 1883 to 1885.

---

A statement has been made in an American medical journal, which, if taken as it is stated, will fill many minds with alarm. "Pure water does not exist in nature," says the writer. Further to reassure us we learn the imperfections of all chemical methods of purification in the same pages. Putting the extreme criticism of the writer aside, those who boil their water may feel reasonably sure that they are running no risks from this course.

---

Dwellers in our hop-growing counties will be glad to learn that the "pickers" will gradually come more and more under supervision. The recently-formed Hop-Pickers' Protection Association is taking upon itself an inquiry into sleeping accommodation for these birds of passage, and other matters concerning their welfare. The flock, which numbers thousands, is far from a welcome one to the inhabitants of the hop-counties, and any means by which their habits are improved will not only benefit the pickers themselves, but the community at large. The task undertaken by the association will, we fancy, not be an easy one, as must be apparent to all travellers on lines traversed by these hop-gatherers; their erratic modes of travelling alone make them somewhat of a care to the railway officials on their route.

---

The subject of teeth is one that appeals to us all. There is no doubt of the deterioration of teeth in modern times. Our own troubles and the remarkable soundness of teeth found in skulls of earlier periods alone proves this. The Americans are perhaps the greatest sufferers, consequently they are provided with the best dentists. Amongst the more civilised peoples a gradual structural and anatomical change is taking place. Those who give proper exercise to the teeth, and live healthy and hardy lives, escape, have comparative immunity from dental troubles, and, as an American authority (Dr. Hugo) summarises: "Man owes the deterioration of his teeth to a change in the mode of life, to change of diet, to reduced physical vigour, to luxury, indolence, vice, sleep robbing, and generally to the sins of omission and commission against hygiene."

